# Health Status Assessment of Diesel Engine Valve Clearance Based on BFA-BOA-VMD Adaptive Noise Reduction and Multi-Channel Information Fusion

**DOI:** 10.3390/s22218129

**Published:** 2022-10-24

**Authors:** Yangshuo Liu, Jianshe Kang, Liang Wen, Yunjie Bai, Chiming Guo

**Affiliations:** 1 Department of Equipment Command and Management, Shijiazhuang Campus, Army Engineering University of PLA, Shijiazhuang 050003, China; 2 66029 Unit of the Chinese People’s Liberation Army, Xilinguolemeng 011200, China

**Keywords:** adaptive noise reduction, condition monitoring, diesel engine, deep neural network, health status assessment, multi-sensor acquisition system, multi-channel information fusion

## Abstract

Regarding the problem of the valve gap health status being difficult to assess due to the complex composition of the condition monitoring signal during the operation of the diesel engine, this paper proposes an adaptive noise reduction and multi-channel information fusion method for the health status assessment of diesel engine valve clearance. For the problem of missing fault information of single-channel sensors in condition monitoring, we built a diesel engine valve clearance preset simulation test bench and constructed a multi-sensor acquisition system to realize the acquisition of diesel engine multi-dimensional cylinder head signals. At the same time, for the problem of poor adaptability of most signal analysis methods, the improved butterfly optimization algorithm by the bacterial foraging algorithm was adopted to adaptively optimize the key parameter for variational mode decomposition, with discrete entropy as the fitness value. Then, to reduce the uncertainty of artificially selecting fault characteristics, the characteristic parameters with a higher recognition degree of diesel engine signal were selected through characteristic sensitivity analysis. To achieve an effective dimensionality reduction integration of multi-channel features, a stacked sparse autoencoder was used to achieve deep fusion of the multi-dimensional feature values. Finally, the feature samples were entered into the constructed one-dimensional convolutional neural network with a four-layer parameter space for training to realize the health status assessment of the diesel engine. In addition, we verified the effectiveness of the method by carrying out valve degradation simulation experiments on the diesel engine test bench. Experimental results show that, compared with other common evaluation methods, the method used in this paper has a better health state evaluation effect.

## 1. Introduction

As the main power core of large mechanical equipment, the diesel engine is widely used in transportation, industrial production, agricultural machinery, the chemical industry, national defense and military equipment, and other fields [1,2,3]. Whether the diesel engine can work normally and healthily often directly affects the normal operation of the entire equipment system. Therefore, it is of great significance to carry out effective condition monitoring and health assessment for diesel engines. In engineering practice, with the prolongation of service time, the valve spring of a diesel engine may gradually deteriorate and deform, and the valve will wear and deposit carbon, which will lead to an abnormal increase of valve clearance, will reduce the efficiency of cylinder flow control, and will then cause the power of diesel engine to decrease. At the same time, the continuous abnormal increase of valve clearance may also cause vicious failures such as cylinder impact or valve breakage, causing huge economic losses and even threatening personal safety [4,5]. In the actual state monitoring process, it is very difficult to directly measure the valve clearance of the diesel engine, and the timeliness is low. The vibration signal of the cylinder head of the diesel engine contains rich state information brought by the inertial impact in the working cycle and various random excitations [6,7]. Therefore, in this study, the vibration signal of the diesel engine cylinder head was collected and its health status was monitored. The process of diesel engine health status assessment mainly includes signal monitoring, data preprocessing, feature extraction, and health status recognition.

The first is the aspect of signal monitoring; most of the current monitoring of vibration signals of diesel engine cylinder heads usually only have a single sensor dimension. A large number of studies have shown that, in the state monitoring and state identification of equipment, multi-dimensional signal monitoring can often achieve better evaluation results than single-dimensional monitoring. Pan et al. [8] conducted an effective evaluation of the performance degradation process of wind turbine gearboxes based on multi-sensor fusion data. Dong et al. [9] achieved a high-precision health status diagnosis and a prediction of hydraulic pump equipment based on a hidden semi-Markov model by collecting multi-sensor signals of the hydraulic pump. Kamal Jafarian et al. [10] used vibration data captured by four sensors placed at different positions on the car engine and in different experimental environments to investigate engine failures, including misfire and valve clearance failures, and combined time-frequency domain analysis methods and neural networks to realize the engine failure mode diagnosis. In this regard, this paper built a multi-sensor monitoring system, conducted a simulated degradation experiment of diesel valve clearance, and collected multi-dimensional cylinder head vibration signals, covering the health status information of the diesel engine from multiple dimensions.

Further, due to the complex structure of the diesel engine, the vibration signal of the cylinder head incorporates the vibration excitation of the whole engine, showing strong non-stationarity and nonlinearity. Therefore, the effective processing and analysis of the vibration signal of the diesel engine cylinder head are also one of the difficulties in its state evaluation process. In this regard, scholars have carried out effective and feasible research. Wei et al. [11] used time synchronization and least squares polynomial fitting to preprocess the original signal and obtained the diesel engine combustion noise transfer function according to the motor test and different injection strategies. Xi et al. [12] used the Stockwell transform to construct a time-frequency reference signal to guide the separation process of kernel-independent component analysis (ICA) to avoid artificial uncertainties in ICA. Shao et al. [13] combined the advantages of the manifold learning algorithm to process nonlinear data and carried out diversified preprocessing on the vibration signal of a marine diesel engine, which greatly improved the quality of feature extraction. Although the above research has achieved certain results, it cannot effectively self-adaptively separate and de-noise the status information and noise interference components in the diesel engine cylinder head signal. In this regard, Wang et al. [14] used a new adaptive wavelet packet threshold function for vibration signal denoising, which can extract truly physically meaningful components from the signal. Wang et al. [15] combined power spectral entropy and variational mode decomposition (VMD) to adaptively process the vibration signal of the diesel engine and used Rihaczek distribution to obtain the time-frequency representation of the diesel engine with high aggregation. Liu et al. [16] proposed an adaptive Wigner-Ville distribution (WVD) and an improved fast correlation filter (FCBF) to solve the cross-term interference of WVD and the redundant control problem of fast FCBF, which effectively separated the vibration signal of the diesel engine’s redundant interference information. Inspired by the above research, we first used the bacterial foraging algorithm (BFA) to improve the optimization performance of the butterfly optimization algorithm (BOA); then, the key parameters α and K in the VMD were optimized based on discrete entropy to realize the adaptive noise reduction of the vibration signal of the diesel engine cylinder head.

In the aspect of signal feature extraction, the time-frequency characteristic analysis of the signal can show the effective state components in the equipment signal from the perspective of the excitation coupling mechanism. Tao et al. [17] used the time-domain statistical method to extract the time-domain features of the signal and obtained high-precision time-frequency domain features through high-resolution multi-synchronous compression transformation. Ahmad Taghizadeh-Alisaraei et al. [18] used time-frequency analysis methods such as short-term Fourier transform (STFT), Wigner-Ville distribution (WVD), and Choi-Williams distribution (CWD) to examine in detail the vibrations produced by a faulty engine. By comparing the time-frequency domain parameters of the vibration response of the normal and faulty injectors, the faulty injectors are effectively detected. Qin et al. [19] combined the extracted time-domain, time-frequency domain, and hand-crafted time-domain statistical features and fed them into a multi-channel deep Siamese convolutional neural network to resist the influence of environmental noise and operating condition changes on the final diagnosis results. Although the above research methods can effectively realize the feature extraction of diesel engine cylinder head vibration signals, the selection of feature parameters is mostly artificial, which brings greater uncertainty to the accuracy of a diesel engine health status assessment. Therefore, we conducted a characteristic sensitivity analysis on the vibration signal of the cylinder head of the diesel engine and selected the characteristic parameters sensitive to the state of health of the diesel engine valve to improve the accuracy of the evaluation.

On the other hand, the multi-channel sensor signal features have redundant information; thus, the effective dimensionality reduction of the features can improve the accuracy of the state evaluation and reduce a certain amount of calculation. In this regard, Yang et al. [20] introduced class information into the traditional non-negative matrix factorization (NMF) and developed an improved discriminative NMF method to achieve the effective dimensionality reduction of time-frequency images of diesel engine vibration signals. Hou et al. [21] used principal component analysis (PCA) to convert high-dimensional fault samples into low-dimensional samples to reduce the computational complexity for the problem of the high dimensionality of fault samples collected by multi-sensors. Chu et al. [22] proposed a visualization method based on texture-enhanced block non-negative matrix factorization (TE-BNMF). By performing time-frequency analysis on the vibration signal of the diesel engine cylinder head and then using the block non-negative matrix factorization (BNMF) algorithm to directly reduce the dimension to extract the feature parameters of the generated local binary feature map, the dimension reduction and enhancement of the feature map were realized. Although the above information fusion method plays the role of feature dimensionality reduction, it still cannot achieve the effect of deep fusion dimensionality reduction and effective state feature enhancement for effective information in multi-dimensional feature samples; therefore, the sparse autoencoder (SAE) with a strong data feature dimensionality reduction and representation ability was selected to perform deep dimensionality reduction learning on multidimensional feature data. SAE adds sparse constraints to the mapping process of the original autoencoder, has stronger data reconstruction and learning capabilities, and is widely used in various fields of military and industry (pattern recognition, semantic segmentation, image processing, etc.) [23,24,25]. Further, we stacked two SAEs together to form a stacked SAE (SSAE), which achieves a better feature dimensionality reduction and feature enhancement by increasing the parameter space and mapping calculation. Finally, in terms of health status recognition, since the feature samples after feature fusion are one-dimensional, we built a one-dimensional convolutional network (1DCNN) to achieve effective mapping between diesel engine feature samples and health status. A large number of studies have proven that, compared to traditional machine learning methods, deep networks have stronger data mining and analysis and learning capabilities [26,27]. Therefore, the evaluation method using 1DCNN as the identification model can accurately and effectively realize the health status evaluation of diesel engine valves.

To sum up, this paper proposes a health status assessment of diesel engine valve clearance based on BFA-BOA-VMD adaptive noise reduction and multi-channel information fusion; then, the effectiveness of the proposed diesel engine state of health assessment method is proven by the diesel engine valve clearance preset simulation experiment. The main contributions and innovations of this paper are as follows:

(1) We carried out a multi-dimensional sensor monitoring system, the valve clearance degradation preset simulation experiment of the diesel engine, and the acquisition of the multi-dimensional vibration signal of the diesel engine cylinder head was realized.

(2) The optimization algorithm and the discrete entropy were combined to realize the adaptive noise reduction of the vibration signal of the diesel engine cylinder head, and then, through feature sensitivity analysis, effective sensitive feature parameters were selected.

(3) We used SSAE with the stacked structure to realize the deep dimensionality reduction and feature reconstruction of multi-dimensional feature samples. Finally, the effective state evaluation of the diesel engine valve clearance was realized through the constructed 1DCNN.

The rest of this paper is organized as follows: Section 2 details the relevant theory and evaluation process of health status assessment methods; Section 3 provides the implementation of the diesel valve clearance degradation preset failure experiment and the preparation of the data samples; Section 4 analyzes and discusses the results of the diesel engine clearance condition assessment; finally, Section 5 presents the conclusions of this study and an outlook for future work.

## 2. Methodology

### 2.1. Adaptive Noise Reduction Based on BFA-BOA-VMD

In the process of the VMD decomposition of vibration signals, two parameters, the penalty factor α and the number of decomposition layers K, have a great influence on the effect of VMD decomposition. Therefore, we used the BOA algorithm improved by BFA to adaptively optimize the important parameters in the VMD, with discrete entropy as the standard, to realize the adaptive noise reduction of the vibration signal of the diesel engine cylinder head.

#### 2.1.1. Discrete Entropy

Discrete entropy is a new algorithm used for measuring the complexity of time series that was proposed by Rostaghi and Azami in 2016 [28]; it overcomes the disadvantage that the arrangement entropy does not consider the magnitude of the amplitude and has the characteristics of good stability and fast calculation speed. The discrete entropy calculation process is as follows:

(1) Normalize the sequence $x=\left\{x_{1},x_{2},\cdots x_{N}\right\}$ to $y=\left\{y_{1},y_{2},\cdots y_{N}\right\}$ using the normal distribution function as the nonlinear normalization function. Where *N* is the length of the sequence, *y* is the cumulative distribution value calculated using the mean and standard deviation of the sequence *x* as the values in the normal distribution of the corresponding parameters, and *y* satisfies the condition of y∈(0,1).

(2) Map *y* to integers in the range [1, *c*] by a linear algorithm, and obtain the sequence,

(1) $$z_{j}^{c}=\mathrm{int}(cy_{j}+0.5)$$

where *c* is the number of categories, *j* is the *j*-th point in the sequence, and int is the rounding function.

(3) Compute the embedding vector and scatter pattern $w_{v_{0}}{{}_{v_{1}}}_{\cdots v_{m-1}}(v=1,2,\cdots ,c)$, and compute the probability *P* for all scatter patterns:

(2) $$P(w_{v_{0}}{{}_{v_{1}}}_{\cdots v_{m-1}})=\frac{num(w_{v_{0}}{{}_{v_{1}}}_{\cdots v_{m-1}})}{N-(m-1)d}$$

where $z_{i}^{c}=v_{0}$,$z_{i+d}^{c}=v_{1}$,…,$z_{i+(m-1)d}^{c}=v_{m-1}$; $w_{v_{0}}{{}_{v_{1}}}_{\cdots v_{m-1}}$ is the scatter pattern, $num(w_{v_{0}}{{}_{v_{1}}}_{\cdots v_{m-1}})$ is the number of $z_{i}^{m,c}$’s mapped to scatter patterns, *m* is the embedding dimension, *d* is the time delay, and N−(m−1)d represents the total number of embedding vectors.

(4) Using the definition of information entropy, calculate the original sequence scatter entropy [29].

(3) $$DE(x,m,c,d)=-\sum_{w=1}^{c^{m}}P(w_{v_{0}}{{}_{v_{1}}}_{\cdots v_{m-1}})\mathrm{In}(P(w_{v_{0}}{{}_{v_{1}}}_{\cdots v_{m-1}}))$$

According to the calculation formula of scatter entropy, it can be found that the larger the scatter entropy value, the greater the complexity of the time series; conversely, the smaller the discrete entropy, the smaller the complexity of the time series and the higher the proportion of effective components. According to the entropy value theorem, the smaller the entropy value, the richer the effective information contained in the signal and the higher the signal-to-noise ratio; conversely, the higher the entropy, the more noise interference information in the signal and the lower the signal-to-noise ratio. Therefore, discrete entropy was used in this paper to determine the decomposition parameters of VMD.

#### 2.1.2. BFA-BOA Optimization Algorithm

The butterfly optimization algorithm (BOA) is a new intelligent optimization algorithm proposed by Arora et al. [30] in 2019, which was inspired by the foraging and mating behavior of butterflies. Butterflies sense and analyze odors in the air to determine food sources and potential directions for mating partners.

By observation, butterflies have very accurate judgments about the location of these sources. Additionally, they can identify different scents and perceive their intensity. A butterfly produces a scent of a certain intensity related to its fitness, which means that when a butterfly moves from one location to another, its fitness changes accordingly. When the butterfly senses that another butterfly is emitting more fragrance in the area, it will approach it, a stage known as the global search; alternatively, when the butterfly cannot perceive a scent larger than itself, it moves randomly, a stage called local search.

The fragrance size *f* is expressed according to the stimulus intensity, and its calculation formula is:

(4) $$f=cI^{a}$$

where *I* is the stimulus intensity, which is related to fitness; *a* is the power exponent, and the empirical value is 0.1; *c* is the sensory factor, and the empirical value is 0.01.

The initial stage of the algorithm first randomly generates the position of each individual butterfly, and then, according to Formula (4), each individual butterfly generates fragrance at its respective initial position. Then, the algorithm enters the global search and local search stages. During the global search process, each individual butterfly moves towards the current global optimal position $g^{*}$, which can be expressed as:

(5) $$x_{i}^{t+1}=x_{i}^{t}+(r_{1}^{2}\times g^{*}-x_{i}^{t})\times f_{i}$$

where $x_{i}^{t}$ represents the position vector of the kth butterfly at the *t*-th iteration, which is the individual cognitive flight part; $r_{1}$ is a random number in the range [0, 1]; $f_{i}$ represents the fragrance intensity of the *i*-th butterfly. The update process of local search can be expressed as [31]:

(6) $$x_{i}^{t+1}=x_{i}^{t}+(r_{2}^{2}\times x_{j}^{t}-x_{k}^{t})\times f_{i}$$

where $x_{j}^{t}$ and $x_{k}^{t}$ represent the *k*-th and *j*-th butterflies randomly selected from the solution space; $r_{2}$ is a random number in the range [0, 1]. During the foraging process of individual butterflies, a switch probability P=0.8 is set to switch between conventional global search and dense local search. In each iteration, a random number in the range [0, 1] is used to compare with the switching probability *P* to determine whether the individual butterfly’s foraging mode is global search or local search.

It can be found that, since the transition probability between the global search stage and the local search stage is generally fixed at 0.8, this makes most butterflies update their positions in the way of the global search stage. At the same time, the position update method in the global search phase is mainly to move toward the position with the strongest fragrance. If this update method is followed all the time, the diversity of the population will be affected in a later stage of the algorithm. Therefore, for the entire BOA algorithm, there are problems such as reduced population diversity, repeated search positions, and falling into local optimum in the later stage of the algorithm. In this regard, this study introduces the bacterial foraging algorithm (BFA) [32], which makes it possible for BOA to jump out of the local optimum in the later iteration of the algorithm by using the foraging mechanism of BFA.

The biological basis of the bacterial foraging algorithm is the intelligent performance of Escherichia coli during the foraging process in the human intestine, i.e., the location of the bacteria is continuously updated through the three steps of chemotaxis, reproduction, and dispersal, so that the bacteria tend to be in a nutrient-rich place. Chemotaxis is characterized by the accumulation of Escherichia coli toward nutrient-rich regions, and dispersal is characterized by the departure of Escherichia coli from the original direction of movement due to a stimulus. The advantage-avoiding mechanism of Escherichia coli can be introduced into BOA. Consider a group of swimming Escherichia coli as the design variable in the solution space, regard the nutrient-rich source in the environment as the optimal solution of the problem to be solved, and define the unfavorable stimulus as some kind of satisfaction in the process of solving the optimization problem. In this way, the design variables in the solution space can adjust their motion behavior in time according to the solution situation of the optimal solution during the optimization process to continuously approach the optimal solution of the problem to be solved. The improved BOA algorithm based on bacterial foraging characteristics, if encountering unfavorable stimuli in the evolution process, implements the dispersal operation to move the individual butterfly, which provides the possibility for the algorithm to jump out of the local optimum. There are many ways to define unfavorable stimuli. In this study, they are defined by the process of optimization and solution: if the optimal value found for five consecutive generations changes within 0.01%, the algorithm is considered to be trapped in a local optimum. Satisfying this condition is considered an unfavorable stimulus, and the dispersing operation is performed on the solution variable.

Combined with the BOA algorithm, the adjustment factor *Z* is introduced, and the formula used in the dispersal operation is:

(7) $$x_{i}^{t+1}=x_{i}^{t}+(r_{2}^{2}\times x_{j}^{t}-x_{k}^{t})\times f_{i}-Z$$

The formula used for the chemotaxis operation is:

(8) $$x_{i}^{t+1}=x_{i}^{t}+(r_{2}^{2}\times x_{j}^{t}-x_{k}^{t})\times f_{i}+Z$$

where $Z=c_{3}r_{3}(x_{kd}^{best}(t)-x_{id}(t))$, the meaning of the other parameters is the same as the BOA algorithm Formulas (5) and (6); the subscripts *k* and *d* represent the *k*-th butterfly individual and the *d*-th dimension, respectively, and k≠i. The learning factor $c_{3}$ is used to guide the individual movement of the butterfly and the inheritance weight of the local optimum. The value of $c_{3}$ is adjusted according to the selection of the values of $c_{1}$ and $c_{2}$. It has been verified by many experiments that, in the case of $\min(c_{1},c_{2})/5<c_{3}<\min(c_{1},c_{2})$, better results can be obtained for the algorithm to jump out of the local optimum.

**Figure 1:**
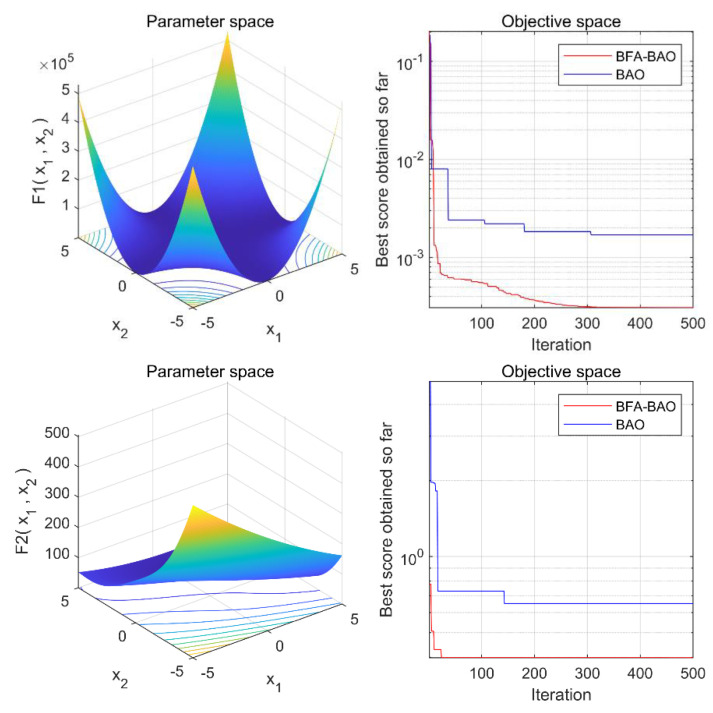

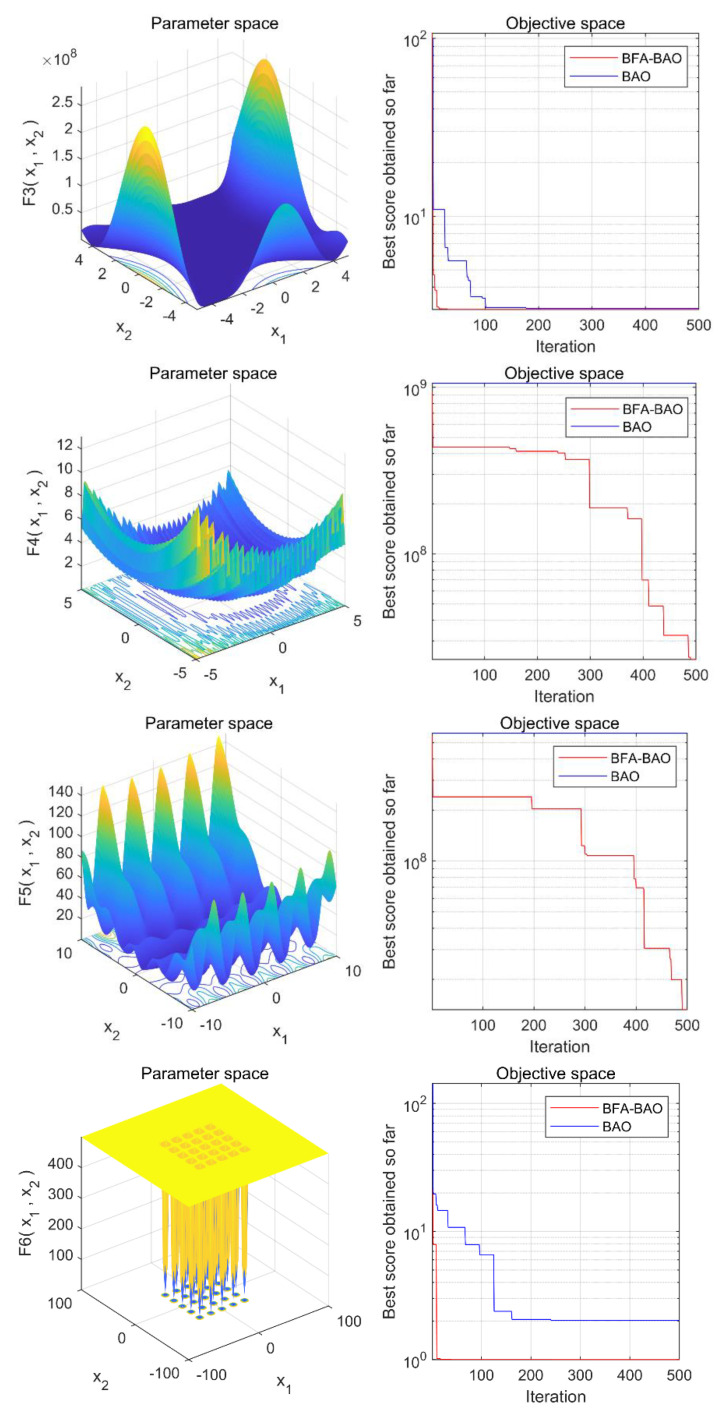
Simulation results of performance test functions on BAO and BFA-BAO algorithms.

To verify the improvement in the optimization ability of the improved BFA-BAO algorithm, we selected six performance test functions to conduct optimization simulation tests on BAO and BFA-BAO, respectively [33,34]. The population size per test and the maximum number of iterations of the algorithm were set to 50 and 500, respectively. The experimental results are shown in Figure 1. From the image of the performance test function, it can be concluded that, in addition to the maximum and minimum values, the test function also has dense and continuous local minima and local maxima, which can effectively test the global optimization ability and search ability of the optimization algorithm. It can be seen from the simulation test results of the BAO and BFA-BAO algorithms of the six types of test functions that the improved BFA-BAO algorithm completed the convergence of the algorithm more quickly and the final fitness function value was lower, i.e., the solution of the BFA-BAO algorithm was closer to the actual minimum value of the test function. As a result, the improved BFA-BAO algorithm had a better global optimization ability and convergence effect than the BFA algorithm.

#### 2.1.3. Adaptive VMD Noise Reduction

**Figure 2:**
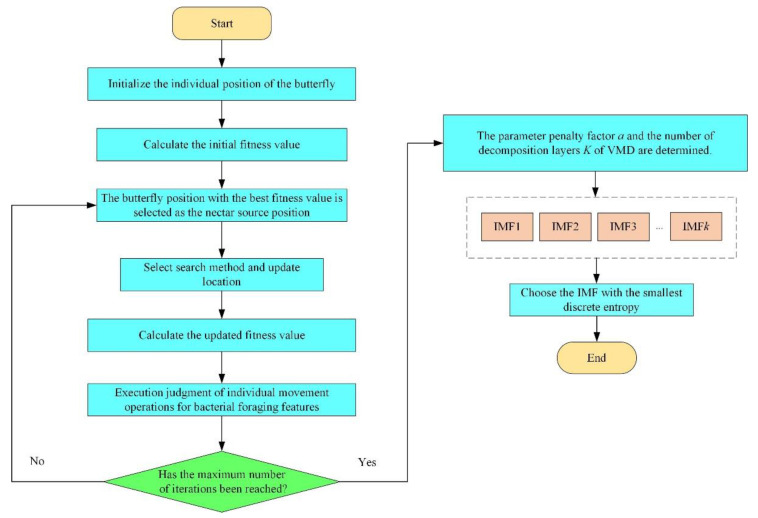
The optimization process of VMD parameter values.

Taking the discrete entropy of the signal as the fitness function, the BFA-BOA optimization algorithm in Section 2.1.2 was used to adaptively optimize the parameter penalty factor *a* and the number of decomposition layers *K* of the VMD. The IMF component with the largest proportion of effective health status information components (the smallest discrete entropy) was selected as the object of subsequent feature sensitivity analysis. The parameter optimization process is shown in Figure 2.

(1) Set the algorithm parameters and initialize the individual position of the butterfly.

(2) Calculate the initial fitness value. Calculate the fitness value of the individual butterfly according to the test function.

(3) Select the nectar source. Arrange the fitness values calculated in step (2) in ascending order, select the butterfly position with the best fitness value as the nectar source position, and calculate its fragrance size.

(4) Location update. Determine whether the current iteration performs a global search or a local search, and then update the position of each butterfly accordingly.

(5) Calculate the fitness value. Calculate the fitness value of the updated position of each butterfly, and update the optimal position.

(6) Individual movement operation based on bacterial foraging characteristics. It is judged whether the unfavorable stimulus is satisfied, i.e., whether the optimal value found for five consecutive generations in the optimization process changes within 0.01%. If so, it is considered to be trapped in a local optimum, and the individual with poor fitness value is subjected to the dispersing operation.

(7) Repeat the iterative process of steps (1) to (6). If the set maximum number of iterations is reached, the algorithm is terminated and the global optimal solution is output, i.e., the IMF component with the lowest global discrete entropy.

### 2.2. Feature Sensitivity Analysis

In the process of feature extraction, human experience selection is often required, which will bring greater uncertainties to the assessment of the health status of the diesel engine, thereby affecting the accuracy of the assessment. Therefore, we used common time-domain and frequency-domain features as a set of characteristic parameters to conduct a characteristic sensitivity analysis on the vibration signal of the diesel engine cylinder head, to select characteristic parameters that are sensitive to the condition of diesel engine valve clearance, and to improve the accuracy and reliability of the evaluation. We used *s* to accurately represent the feature sensitivity:

(9) $$s=\left|\frac{f_{i}-f}{f}\right|$$

where *s* is the sensitivity, $f_{i}$ is the target analysis eigenvalue, and f is the reference eigenvalue. In this paper, the parameter indexes under the normal valve clearance state of the diesel engine were used as the reference eigenvalues, and the parameter indexes under other valve clearance conditions were used as the target analysis eigenvalues. The feature set included 14 common time domain features and 4 common frequency domain features. The 14 time-domain features included 9 dimensioned features: maximum value (*f*_1_), minimum value (*f*_2_), mean value (*f*_3_), mean square value (*f*_4_), peak value (*f*_5_), peak-to-peak value (*f*_6_), absolute mean value (*f*_7_), variance (*f*_8_), and root mean Square (RMS, *f*_9_), and they included 5 dimensionless features: impulse factor (*f*_10_), crest factor (*f*_11_), shape factor (*f*_12_), remainder gap (*f*_13_) and factor margin factor (*f*_14_). The 4 frequency domain characteristic parameters included average frequency (*f*_15_), frequency center (*f*_16_), frequency variance (*f*_17_), and root mean square frequency (*f*_18_) [35,36,37]. The serial numbers and formulas corresponding to the time-domain and frequency-domain characteristic parameters are shown in Table 1 (the time domain signal is represented by $x_{i}$, *N* represents the length of $x_{i}$, $s_{k}$ represents the spectrum of *k*, and $e_{k}$ represents the frequency value corresponding to the spectrum of the *k*-th point).

### 2.3. Deep Fusion of Multi-Channel Feature Values

After the adaptive noise reduction and feature sensitivity analysis of the multi-channel cylinder head vibration signal of the diesel engine, the sensitive features were selected and extracted. This section describes the stacked sparse autoencoder (SSAE) that was built to realize the deep fusion and dimensionality reduction reconstruction of multi-channel feature values so that the feature vectors can be re-expressed in high-level abstraction.

#### 2.3.1. Sparse Autoencoders (SAE)

**Figure 3:**
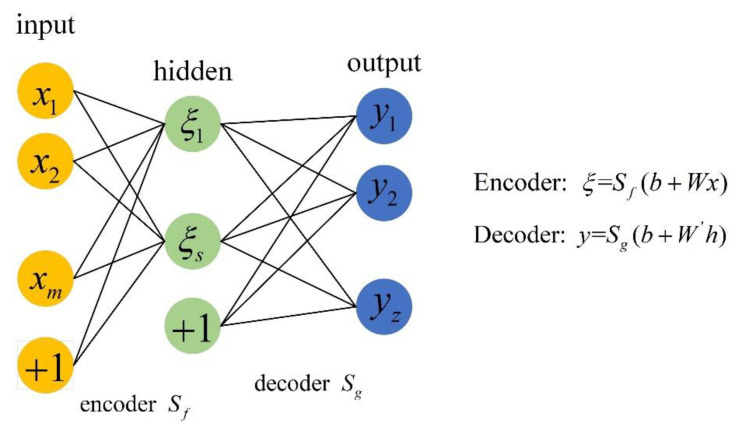
Structure of AE.

An autoencoder (AE) is an unsupervised neural network model that includes an encoder and decoder; it is mainly composed of the input layer, hidden layer, and output layer in the structure (see Figure 3). An AE can map the data to the high-level parameter space to express it abstractly, and the data features can be reconstructed and enhanced. Compared with traditional dimensionality reduction methods, such as principal component analysis (PCA) and independent component analysis (ICA), AE can not only reduce the data dimension, but it can also ensure the integrity and invariance of data features, which ensures the quality of input feature samples for subsequent network training [38].

SAE is consistent with AE in terms of structural composition, but SAE adds a sparsity limit to the loss function of AE; at the same time, only some hidden layer nodes are “active”; thus, the entire AE network becomes sparse. Assuming that the hidden layer activation function uses sigmoid, the hidden layer output is 1 to indicate that the node is “active”, and the hidden layer output is 0 to indicate that the node is “inactive”. Based on this, we introduced *KL* dispersion to measure the similarity between the average activation output of a certain hidden layer node and the sparsity ρ, and we set:

(10) $$K_{L}(\rho ||\overset{\wedge }{\rho_{j}})=\rho \log\frac{\rho }{\overset{\wedge }{\rho_{j}}}+(1-\rho )\log\frac{1-\rho }{1-\overset{\wedge }{\rho_{j}}}$$

(11) $$\overset{\wedge }{\rho_{j}}=\frac{1}{m}\sum_{i=1}^{m}a_{j}(x_{i})$$

where $\overset{\wedge }{\rho_{j}}$ is the average sparse activation, $x_{i}$ is the training sample, and *m* is the number of training samples; $a_{j}(x_{i})$ is the response output of the *j*-th node of the hidden layer to the *i*-th sample. In general, the sparsity coefficient ρ is set to 0.05 or 0.1. The larger the *KL* divergence, the greater the difference between ρ and $\overset{\wedge }{\rho_{j}}$, and the *KL* divergence equal to 0 means the two are completely equal. Then, the *KL* dispersion is added as a regular term to the loss function of AE to constrain the sparse rows of the entire AE network [39,40]:

(12) $$J_{SAE}(W,b)=J_{AE}(W,b)+\beta \sum_{j=1}^{m}K_{L}(\rho ||\overset{\wedge }{\rho_{j}})$$

where β is the weight coefficient of the sparse constraint.

#### 2.3.2. Deep Fusion of Multi-Channel Feature Values Based on SSAE

**Figure 4:**
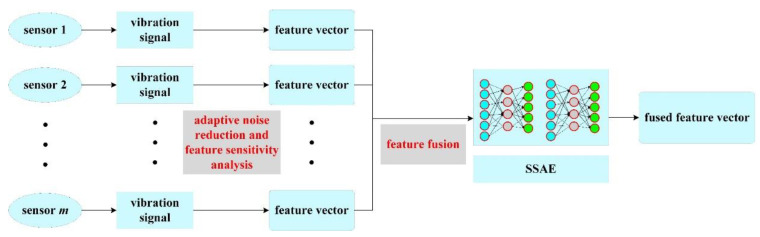
Schematic diagram of the multi-channel feature values fusion process.

Further, on the basis of SAE, we used a stacking method to form two SAE networks into a stacked SAE network (SSAE). Because SSAE has a deeper network structure and parameter space, it has greater improvements and advantages than SAE in data fusion and feature reconstruction. In the specific process of multi-channel feature values fusion, assuming that there are *m* signal acquisition channels, after the vibration signal of the diesel engine cylinder head is subjected to adaptive noise reduction and characteristic sensitivity analysis, *n* characteristic parameters are selected. Then, the different channel features are concatenated and normalized so that the dimension of the feature vector before dimensionality reduction is m×n×1. Further, the feature output dimension after dimension reduction is determined to be *l* according to the number of hidden layer network nodes of SSAE. Then, the cylinder head vibration signal under each valve clearance state can finally obtain a feature sample with dimension l×1. The multi-channel feature values fusion process is shown in Figure 4; after the multidimensional sensor signal is analyzed by adaptive noise reduction and feature sensitivity, the effective dimensionality reduction of multi-source feature vectors can be realized through the SSAE network to construct feature samples.

### 2.4. One-Dimensional Convolutional Neural Network (1DCNN)

Convolutional neural network (CNN) is a type of typical feedforward neural network with a deep structure that includes convolution calculation. As a type of deep learning model, it is widely used in various fields such as image recognition and speech recognition. Its classic network structure models include LetNet-5 model, AlexNet network, GoogleNet network, and VGG network. The basic structure of CNN usually consists of an input layer, a convolution layer, a pooling layer, a fully connected layer, and an output layer [41,42]. The CNN network maps the data input into the high-dimensional nonlinear parameter space through the convolution calculation of the convolution layer, the dimensionality reduction feature mapping of the pooling layer, and the classification feature recognition of the fully connected layer to realize the deep learning and recognition of the data.

**Figure 5:**
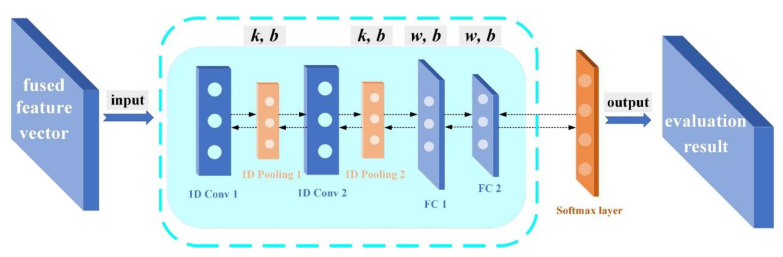
The network structure of 1DCNN.

According to the input dimension of CNN, input samples can be divided into two-dimensional image samples and one-dimensional matrix samples. Since the feature samples processed in Section 2.3 are one-dimensional matrices, a one-dimensional convolutional neural network (1DCNN) was conducted in this paper to evaluate the state of diesel engine valve clearance. The 1DCNN consisted of four network layers, including two convolutional layers and two fully connected layers (only the network layers with parameter weights are calculated), and the network structure of 1DCNN is shown in Figure 5. After the multi-level operation of the convolutional layer, pooling layer, and fully connected layer, the characteristic samples constructed in this paper can finally obtain the prediction label of the classification layer and complete the status evaluation. The structural parameter settings of the network are given in the network training part of the experimental verification.

### 2.5. Diesel Engine Valve Clearance State Assessment Flow

**Figure 6:**
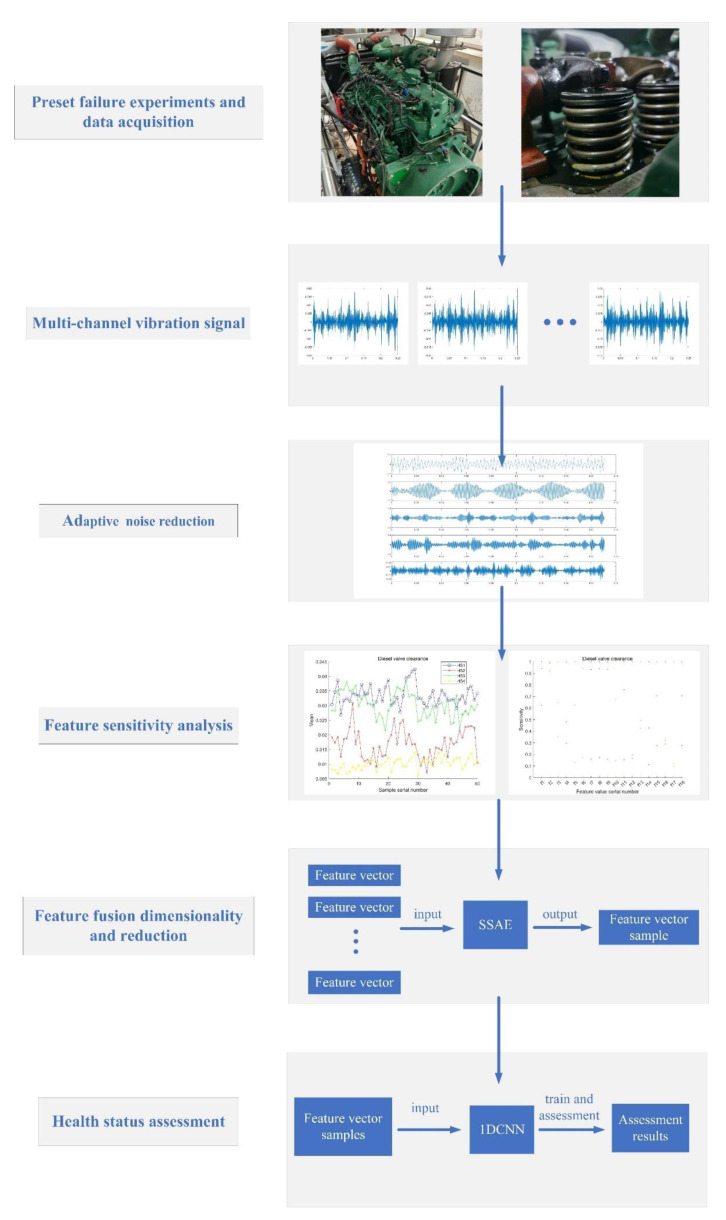
The flowchart of the proposed diesel engine valve clearance state assessment method.

Figure 6 shows the flow of the diesel engine valve clearance state assessment method proposed in this paper.

(1) The preset simulation experiment of diesel engine valve clearance degradation and multi-channel sensor data acquisition of the cylinder head vibration signal are carried out;

(2) Adaptive noise reduction is performed on the vibration signal of the cylinder head, and the target IMF component is selected according to the discrete entropy standard;

(3) According to the time domain and frequency domain feature sets, the characteristic sensitivity analysis of the target IMF components under different valve clearance states is carried out, and the characteristic parameters that are most sensitive to the valve clearance states are selected to form the eigenvectors;

(4) The multi-channel feature vector is input into the SSAE network for deep fusion and dimensionality reduction, and the feature samples are obtained that are used for the network input;

(5) The feature samples are divided into a training set and test set according to the proportion, and they are input into 1DCNN to realize the training and assessment of the health status of diesel engine valve clearance.

## 3. Case Study

### 3.1. Preset Failure Experiments and Data Collection

**Figure 7:**
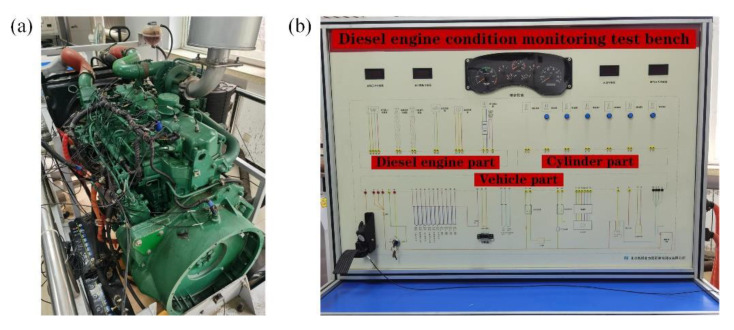
Diesel engine system: (**a**) diesel engine; (**b**) control panel.

**Figure 8:**
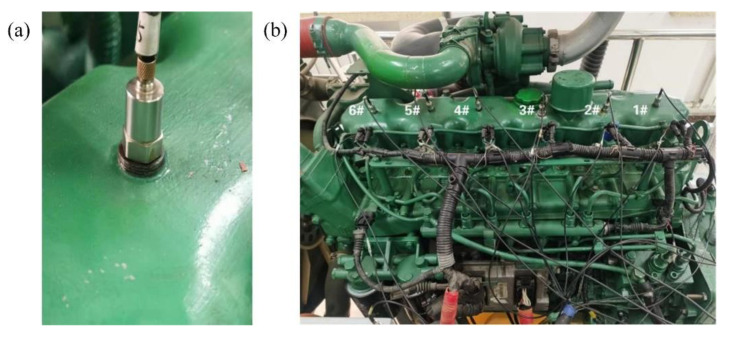
Vibration acceleration sensor and its installation location: (**a**) vibration acceleration sensor; (**b**) sensor installation location.

This paper relied on the diesel engine condition monitoring test bench to carry out the valve clearance simulation degradation experiment to verify the effectiveness of the method proposed in this paper. The test bench system was mainly composed of a diesel engine and a control panel, as shown in Figure 7; the ignition, flameout, and power output of the diesel engine can be controlled through the control panel. The model of the diesel engine is a six-cylinder high-pressure common rail diesel engine, and its basic parameters are shown in Table 2. To realize the multi-channel acquisition of the vibration signal of the diesel engine cylinder head, we installed six acceleration vibration sensors in parallel on the cylinder head surface of the diesel engine, and the fixing method was glue. The vibration acceleration sensor and its installation location distribution are shown in Figure 8.

**Figure 9:**
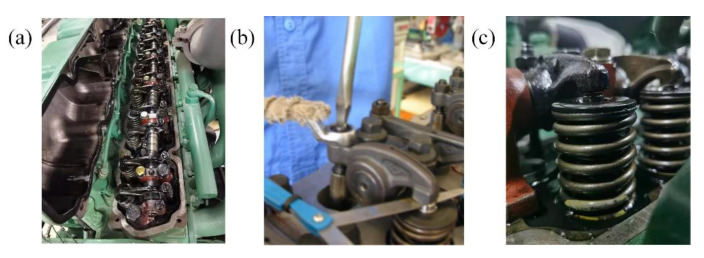
Valve clearance adjustment process: (**a**) cylinder head is removed; (**b**)a valve clearance is adjusted; (**c**) the adjusted valve clearance.

Considering that the valve clearance increase is the main problem in engineering practice, different valve clearance sizes of the intake valve were preset to simulate the valve clearance increase process caused by valve degradation. The valve clearance of the diesel engine was adjusted by the feeler gauge to simulate the abnormal increase of clearance in the process of valve degradation. In the process of adjusting the valve clearance, the cylinder head of the diesel engine was first disassembled, and then the valve clearance adjustment cylinder was selected. In the experiment, the third cylinder was set as the preset simulation cylinder. The adjustment process of the valve clearance is shown in Figure 9.

To adjust the valve clearance when the diesel engine is cold, the lock nut on the upper part of the third cylinder valve needs to be loosened, and the adjusting screw needs turned with a screwdriver. The healthy valve clearance of a diesel engine is generally: intake valve is 0.25 mm~0.3 mm, exhaust valve is 0.3 mm~0.35 mm. This experiment simulated the valve clearance degradation process with a total of four preset health states, namely normal (healthy), slightly increased valve clearance (general), increased valve clearance (attention), and severely increased valve clearance (deteriorated); the settings are shown in Table 3.

During the acquisition of vibration signals, the sampling frequency was 20 kHz, the single sampling time was 10 s, and the sampling interval was 15 s. The format of the vibration signal data of the diesel engine cylinder head initially collected was “.tdms”. We then converted it to the format of “.mat” in the Matlab software for further processing. For the vibration signal under each valve clearance state, we took 5000 points as 1 sample, and each took 54 samples.

### 3.2. Preparation of Feature Samples

#### 3.2.1. Adaptive Noise Reduction

Firstly, adaptive noise reduction was performed on the cylinder head vibration signal under different valve clearance states. In the VMD decomposition process, the value range of the penalty factor *a* is generally 1.5~2 times the number of sample points, i.e., [7500, 10,000], and the value range of the decomposition level *K* is generally [2, 7]. The number of individual butterflies and the number of iterations in the BFA-BOA algorithm were set to 20 and 100, respectively. We uniformly sampled the six-channel data under the same valve clearance state, respectively, and Table 4 lists the parameter averages of the optimization results. After adaptive noise reduction, the IMF component with the smallest discrete entropy was selected for each vibration signal sample for the next step of feature sensitivity analysis.

#### 3.2.2. Sensitive Feature Selection

**Figure 10:**
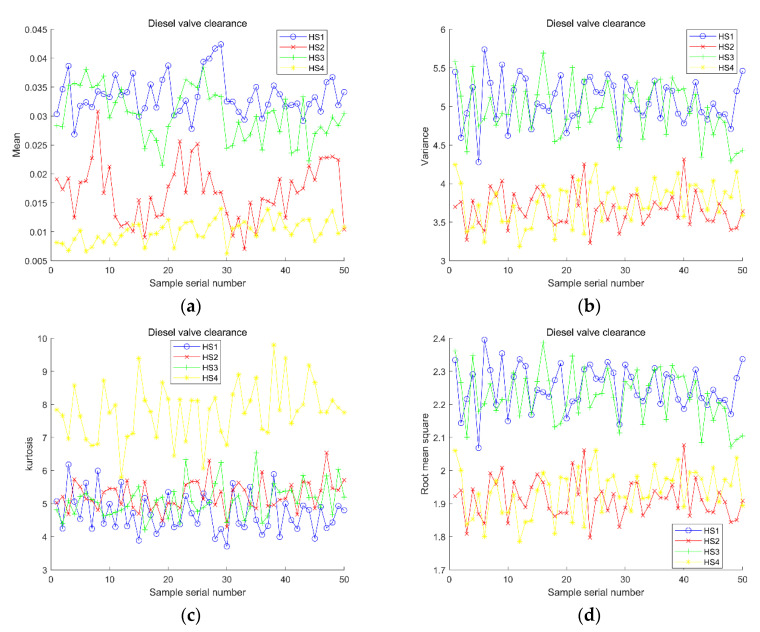
Variation trend graph of characteristic parameters. (**a**) Mean; (**b**) Variance; (**c**) Kurtosis; (**d**) Root mean square.

**Figure 11:**
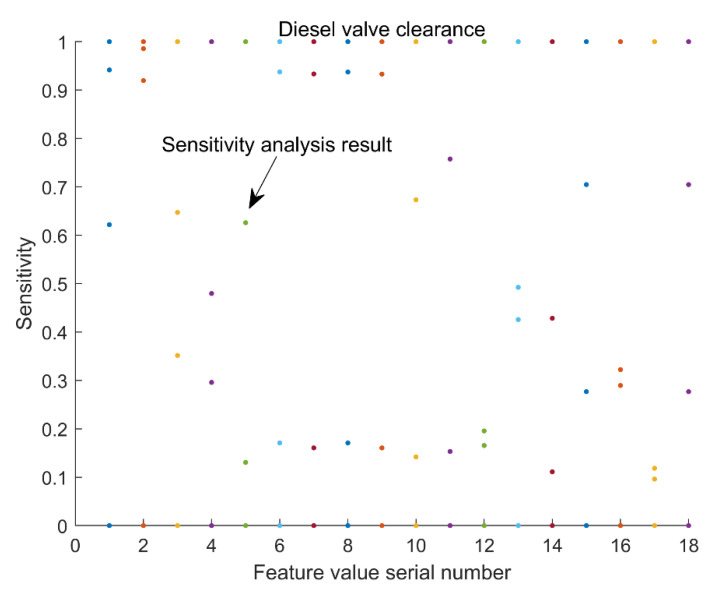
Sensitivity analysis of the feature set.

A total of 50 groups of samples were selected from four kinds of valve clearance health status IMF samples for feature sensitivity analysis. Due to space limitations, only the analysis results of mean, variance, kurtosis, and root mean square are listed here (see Figure 10). From the change trend results of the characteristic parameters in Figure 10, it can be concluded that some characteristics cannot well distinguish the health status of the valve clearance of the diesel engine. Therefore, we used the feature sensitivity *s* in Section 2.2 as the standard and selected the top 14 time-domain and frequency-domain feature parameters with higher sensitivity to improve the accuracy of the state evaluation. After obtaining the sensitivity *s*, the sensitivities of the IMF components of all valve clearance states were normalized according to the direction of the characteristic parameters, and the results are shown in Figure 11. As can be seen from the results of Figure 11, the 18 different types of time and frequency domain eigenvalues can distinguish the four states of the valve gap to a certain extent. However, it is still not possible to quantitatively select the more sensitive feature sets. Therefore, we further summed up and sort the sensitivity s, and the results are shown in Table 5; the top 14 characteristic parameters with the highest sensitivities were selected as the feature extraction objects of the IMF, and the feature vector was constructed. From the results in Table 5, it can be concluded that the selected characteristic parameters are: *f*_2_, *f*_1_, *f*_6_, *f*_8_, *f*_7_, *f*_9_, *f*_3_, *f*_18_, *f*_15_, *f*_13_, *f*_11_, *f*_10_, *f*_4_, *f*_5_. In this way, each IMF component sample can obtain a 14×1-dimensional feature vector.

#### 3.2.3. Muti-Channel Feature Vectors Fusion

After the feature analysis and extraction in Section 3.2.1, a 14×1-dimensional feature vector was obtained for the six-channel data under each valve clearance state. We first concatenated and normalized these six feature vectors to obtain a 84×1-dimensional feature vector. Then, the concatenated feature vectors were input into the SSAE network for fusion and dimensionality reduction. The hyperparameter settings of SSAE are shown in Table 6.

It can be seen from the hyperparameter settings in Table 6 that the number of hidden layer nodes of the second layer of SAE is 25; thus, the dimension of the feature vector after fusion and dimension reduction was 25×1. Finally, 54 25×1-dimensional eigenvector samples can be obtained for each diesel engine valve clearance health state.

## 4. Health Status Assessment Using 1DCNN

**Figure 12:**
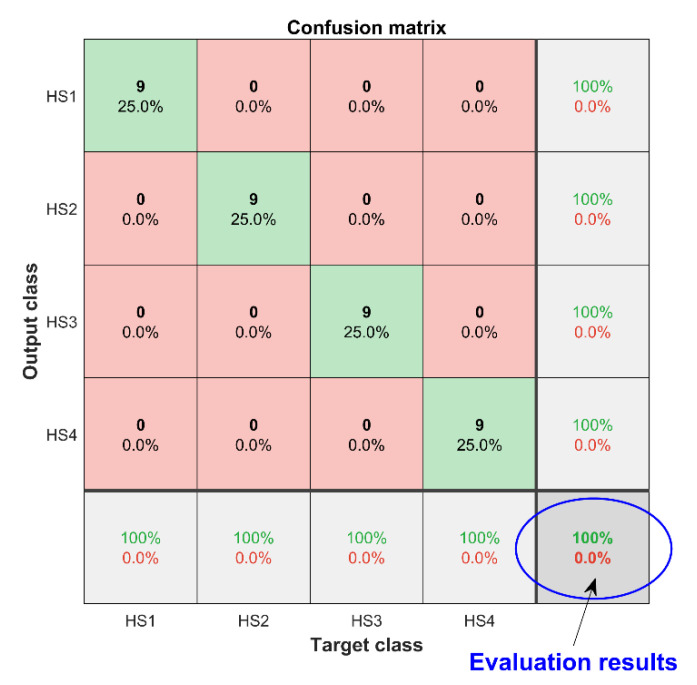
Confusion matrix of diesel valve clearance health assessment results.

The diesel valve clearance feature samples constructed in Section 3 were divided into a training set and a test set with a ratio of 5:1, i.e., the number of training samples was 180 and the number of test samples was 36. The training samples were input into 1DCNN for training, and the structural parameter settings of 1DCNN are shown in Table 7. Using the trained 1DCNN to evaluate the health status of the test samples, the accuracy of the 10 evaluation results was 100%, and the confusion matrix of the evaluation results is shown in Figure 12. It can be concluded that the state-of-health evaluation method for diesel engine valve clearance can achieve high evaluation accuracy and effect, which proves the effectiveness of the evaluation method.

## 5. Results Analysis and Method Comparison

### 5.1. Small Sample Analysis

To verify the effectiveness and stability of the evaluation method in this paper under the condition of small samples, we set the total number of samples to 216, 192, 168, 144, and 120, respectively, and the corresponding training set, test set number, and evaluation accuracy are shown in Table 8.

**Figure 13:**
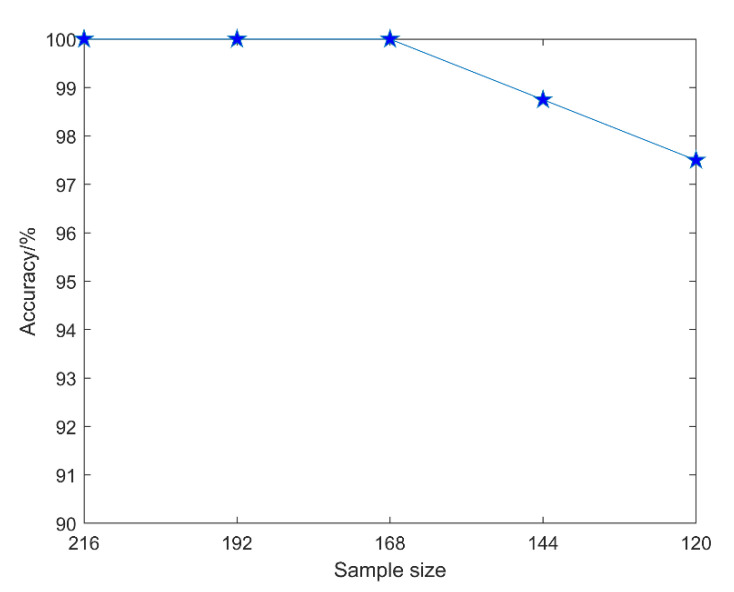
Graph of changes in evaluation results for different sample sizes.

From the variation trend of the evaluation accuracy with the sample size in Figure 13, it can be clearly seen that the evaluation method in this paper did not have a large fluctuation when the sample size was reduced, and it can still maintain a high evaluation accuracy. Even if the number of training sets is only 100, the average evaluation accuracy can reach 97.5%. This demonstrates the effectiveness and small-sample robustness of our evaluation method.

### 5.2. Comparison Analysis

#### 5.2.1. Optimization Algorithm Comparison Analysis

**Figure 14:**
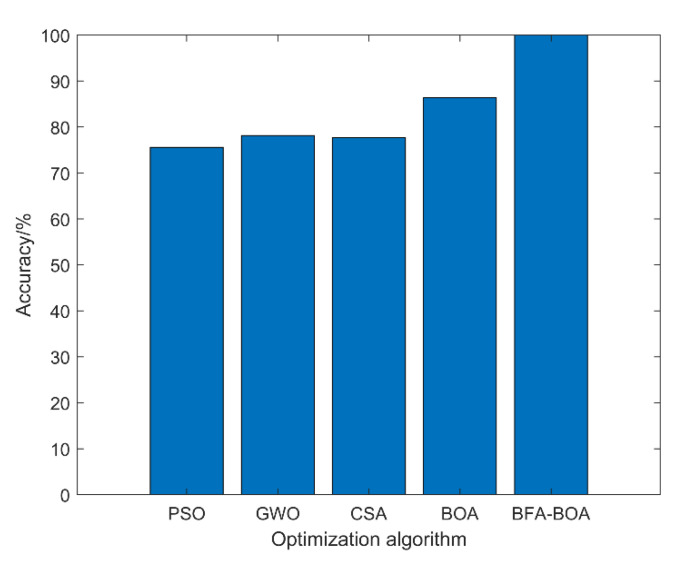
Graph of the evaluation results under different optimization algorithms.

To verify the effectiveness of the BFA-BOA-based adaptive VMD noise reduction method in the signal preprocessing stage, in this section, common swarm optimization algorithms, including the particle swarm optimization algorithm (PSO) [43], gray wolf optimization algorithm (GWO) [44], and cuckoo search algorithm (CSA) [45], are used to carry out the adaptive noise reduction of the signal. At the same time, the separate BOA algorithm was compared to verify the effectiveness of the BFA-BOA algorithm that considers the BFA stimulus in this paper. The rest of the evaluation process was consistent with this paper, and the optimization ability of different algorithms was determined by the final evaluation accuracy. The average of the 10 evaluation results is shown in Table 9 and Figure 14.

As can be seen from the experimental results in Table 9 and Figure 14, compared with the common group optimization algorithms such as PSO, GWO, and CSA, the improved BFA-BOA optimization algorithm proposed in this paper can more effectively realize the adaptive noise reduction of the vibration signal of the diesel engine cylinder head; thus, a very high state evaluation accuracy can be obtained. In addition, the evaluation effect of using the BOA algorithm alone is worse than that of the BFA-BOA algorithm, which once again proves that the global optimization ability of BOA has been greatly improved after considering the stimulus factor of BFA.

#### 5.2.2. Method Comparison Analysis

**Figure 15:**
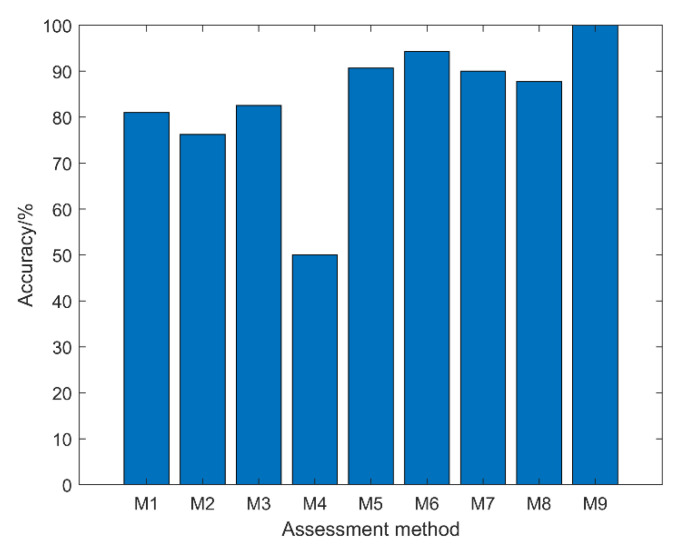
Graph of the evaluation results under different assessment methods.

To further demonstrate the effectiveness of the health status assessment method proposed in this paper, the method was compared with common health status assessment methods. These methods include: (1) The vibration signal of the diesel engine cylinder head was decomposed by VMD with unified parameters, i.e., the value of the penalty factor *a* is 8000, and the value of the decomposition level *K* is 3. Then, the last IMF component for feature parameter extraction was selected, and the other processes were consistent with the method in this paper (M1). (2) Only adaptive VMD decomposition was performed on the vibration signal without feature fusion based on SSAE. The parameter settings and other processes are consistent with the method in this paper (M2). (3) The main analytic hierarchy process (PCA) was used to fuse the dimensionality reduction of the feature vector. In the dimensionality reduction calculation process, the first few eigenvalues with a cumulative contribution value of more than 95% were selected as the characteristic samples for training, and the other processes are consistent with the method in this paper (M3) [46]. (4) The one-dimensional vibration signal was directly input into the one-dimensional 1DCNN constructed in this paper for training evaluation (M4), and the timing signal was selected for the first channel. (5) The feature samples constructed in this paper were directly input into the support vector machine (SVM) for training and evaluation (M5). Among them, the combination of the four key parameters of SVM, weight coefficient, penalty factor, radial base kernel function parameters, and insensitive loss function parameters were [0.08, 2.80, 0.58, 0.01] [47]. (6) The feature samples constructed in this paper were directly input into the Softmax layer with a classification function for evaluation (M6) [48]. The parameter settings and other processes are consistent with the method in this paper. (7) The feature samples constructed in this paper were directly input into the long short-term memory networks (LSTM) for evaluation (M7) [49]. (8) The feature samples constructed in this paper were directly input into the deep belief networks (DBN) for evaluation (M8) [50]. The method in this paper was denoted as (M9), and the total sample size was chosen as 216. The average evaluation results of 10 experiments are shown in Table 10 and Figure 15.

From the evaluation results in Table 9 and Figure 14, the following conclusions can be drawn: (1) Since the M1 method fails to adaptively optimize its key parameters when the diesel engine cylinder head vibration signal is decomposed by VMD, the noise component and the effective health status component of the signal cannot be effectively separated; thus, the evaluation accuracy is lower than that of the method in this paper. (2) The M2 method does not fuse the feature vectors, resulting in redundant information between multi-channel signals drowning out the effective health status information, while the M3 method achieves the dimensionality reduction of PCA but fails to deeply reconstruct and enhance the data features. Therefore, the evaluation accuracy of the M2 and M3 methods is lower than that of the method in this paper. (3) The M4 method directly inputs the one-dimensional vibration signal into the 1DCNN network. Since there are many noise interference components in the signal without noise reduction and feature extraction, the signal-to-noise ratio of the effective component is very low. Therefore, a good mapping relationship cannot be formed between the input and the two ends of the evaluation result, resulting in a poor evaluation result. (4) Although the M5 and M6 methods achieve an effective evaluation of fused feature samples, the accuracy rate also reaches more than 90%. However, since neither SVM nor Softmax layer have the powerful parameter space and nonlinear mapping learning ability of 1DCNN, the evaluation effect of these two methods is still lower than that of the method in this paper. (5) Due to the small sample size of the features in this paper and because the parameter space of the LSTM and DBN networks is too large, the network cannot be fully trained, which leads to the evaluation accuracy being lower than the method in this article.

To sum up, compared with other common state of health evaluation methods, the proposed method for evaluating the state of health of diesel engine valve clearance has good accuracy and superiority.

## 6. Conclusions

The valve train is one of the key components of a diesel engine. If the valve clearance of the diesel engine continues to increase abnormally, it may cause serious failures such as cylinder collision or valve breakage, and it will also threaten the safety of operators. Based on this situation, this paper proposed a health status assessment of diesel engine valve clearance based on BFA-BOA-VMD adaptive noise reduction and multi-channel information fusion, which effectively improved the accuracy of the diesel engine valve gap health state assessment. By carrying out the simulation experiment of diesel engine valve clearance degradation, the vibration signal of the multi-channel diesel engine cylinder head was collected, and the BFA-BOA-VMD method was used to realize the adaptive noise reduction of the signal and the selection of effective IMF components, which improved the signal-to-noise ratio of the signal. Further, the characteristic sensitivity analysis selected the time-domain and frequency-domain characteristic parameters that are sensitive to the state of health of the diesel valve clearance. Feature fusion and dimensionality reduction based on SSAE network further reconstructed and enhanced data features, highlighting state information. Finally, we conducted health status assessment experiments on the diesel engine valve gap preset experimental data set. Experimental results showing that, compared with the common state assessment algorithm, the proposed method has good evaluation accuracy and superiority.

To sum up, the method for assessing the health status of diesel engine valve clearances proposed in this paper, as an important technical means in equipment health management, has very important application prospects and significance and also provides new theoretical support and ideas for equipment health management and assessment. In future research, more effective signal noise reduction methods, feature fusion methods with stronger reconstruction capability, and deep network models with stronger learning abilities can be further studied.

## Figures and Tables

**Table 1:** The formulas correspond to the characteristic parameters.

Number	Characteristic Parameters	Formula	Number	Characteristic Parameters	Formula
1	*f*_1_	$f_{1}=\max(x_{i})$	10	*f*_10_	$f_{10}=\frac{f_{4}}{f_{11}}$
2	*f*_2_	$f_{2}=\min(x_{i})$	11	*f*_11_	$f_{11}=\frac{f_{3}}{f_{4}}$
3	*f*_3_	$f_{3}=\frac{1}{N}\sum_{i=1}^{N}x_{i}$	12	*f*_12_	$f_{12}=\frac{f_{3}}{f_{11}}$
4	*f*_4_	$f_{4}=\frac{1}{N}\sum_{i=1}^{N}x_{i}^{2}$	13	*f*_13_	$f_{13}=\frac{f_{3}}{{\left(\frac{\sum_{i=1}^{N}\sqrt{\left|x_{i}\right|}}{N}\right)}^{2}}$
5	*f*_5_	$f_{5}=\max\left|x_{i}\right|$	14	*f*_14_	$f_{14}=\frac{f_{3}}{f_{9}}$
6	*f*_6_	$f_{6}=f_{1}-f_{2}$	15	*f*_15_	$f_{15}=\frac{\sum_{k=1}^{K}s(k)}{K}$
7	*f*_7_	$f_{7}=\frac{\sum_{i=1}^{N}\left|x_{i}\right|}{N}$	16	*f*_16_	$f_{16}=\frac{\sum_{k=1}^{K}e_{k}s(k)}{\sum_{k=1}^{K}s(k)}$
8	*f*_8_	$f_{8}=\frac{1}{N}\sum_{i=1}^{N}{\left(x_{i}-f_{4}\right)}^{2}$	17	*f*_17_	$f_{17}=\sqrt{\frac{\sum_{k=1}^{K}{\left(e_{k}-f_{16}\right)}^{2}s(k)}{\sum_{k=1}^{K}s(k)}}$
9	*f*_9_	$f_{9}=\sqrt{f_{3}}$	18	*f*_18_	$f_{18}=\sqrt{\frac{\sum_{k=1}^{K}e_{k}{}^{2}s(k)}{\sum_{k=1}^{K}s(k)}}$

**Table 2:** Basic parameters of diesel engine.

Category	Parameter	Category	Parameter
Diesel Type	Six-cylinder in-line, high common rail	Rated speed	2300 r/min
Engine model	CA6DF3-20E3	Maximum torque	760 NM
Engine size	1330×970×1005 mm	Net power	147 kW

**Table 3:** Valve clearance presets.

Serial Number	Health Status	Intake Valve Clearance	Exhaust Valve Clearance
HS1	normal status	0.3 mm	0.3 mm
HS2	intake valve clearance slightly increased	0.4 mm	0.3 mm
HS3	intake valve clearance increased	0.5 mm	0.3 mm
HS4	severely increased intake valve clearance	0.7 mm	0.3 mm

**Table 4:** Optimization results for *a* and *K*.

Type	Optimum Value of *a*	Optimum Value of *K*	Minimum Discrete Entropy
HS1	8351	4	3.7115
HS2	8516	4	3.9040
HS3	8623	5	3.4564
HS4	8496	6	3.2938

**Table 5:** The sorting results of feature sensitivity.

Feature	Sum of Sensitivities	Feature	Sum of Sensitivities
*f*_2_	2.9052	*f*_13_	1.9180
*f*_1_	2.5635	*f*_11_	1.9107
*f*_6_	2.1085	*f*_10_	1.8152
*f*_8_	2.1082	*f*_4_	1.7756
*f*_7_	2.0939	*f*_5_	1.7564
*f*_9_	2.0935	*f*_12_	1.6118
*f*_3_	1.9988	*f*_14_	1.5395
*f*_18_	1.9815	*f*_16_	1.3611
*f*_15_	1.9814	*f*_17_	1.2147

**Table 6:** Hyperparameter settings of SSAE.

Layer	Hyperparameter	Value
First−layer SAE	Number of hidden layer nodes	60
	L2 regularization weight decay coefficient	1 × 10^−3^
	Sparse penalty weights	2 × 10^−3^
	Sparsity parameter	1 × 10^−4^
	Decoder transfer function	Purelin
	The maximum number of iterations	100
Second−layer SAE	Number of hidden layer nodes	25
	L2 regularization weight decay coefficient	1 × 10^−3^
	Sparse penalty weights	2 × 10^−3^
	Sparsity parameter	1 × 10^−4^
	Decoder transfer function	Purelin
	The maximum number of iterations	100

**Table 7:** Structural parameter settings of 1DCNN.

Layers	Kernel/Pool/Drop Size	Number	Output Size
Conv1	5	64	(N, 21, 64)
MaxPooling1	2	/	(N, 20, 64)
Conv1	3	64	(N, 18, 64)
MaxPooling2	2	/	(N, 17, 64)
Flatten	/	/	(N, 1088)
FC1	/	256	(N, 256)
Dropout	0.5	/	(N, 256)
FC2	/	128	(N, 128)
Dropout	0.5	/	(N, 128)
Softmax/Classification	/	4	(N, 4)

**Table 8:** Assessment results of different sample sizes.

Total Set	Training Set	Test Set	Assessment Accuracy (%)
216	180	36	100
192	160	32	100
168	140	28	100
144	120	24	98.75
120	100	20	97.50

**Table 9:** Assessment results under different optimization algorithms.

Optimization Algorithms	Assessment Accuracy (%)
PSO	75.58
GWO	78.15
CSA	77.66
BOA	86.37
BFA-BOA	100

**Table 10:** Assessment results under different assessment methods.

Assessment Methods	Assessment Accuracy (%)
M1	81.01
M2	76.21
M3	82.54
M4	50.05
M5	90.66
M6	94.33
M7	90.02
M8	87.74
M9	100

## Data Availability

The study data used in this article can be obtained by contacting the corresponding authors.
